# Effect of Intercritical Quenching Temperature on Microstructure and Mechanical Performance of Cr-Ni-Mo-V Steel with Banded Structure

**DOI:** 10.3390/ma18174017

**Published:** 2025-08-27

**Authors:** Yunfei Du, Xiaosheng Zhou, Rui Bai, Yaqin Zhang

**Affiliations:** 1 Department of Mechanical Engineering, Taiyuan Institute of Technology, Taiyuan 030008, China; bair@tit.edu.cn (R.B.); zhangyaqin2020@163.com (Y.Z.); 2 School of Mechanical Engineering, North University of China, Taiyuan 030051, China; zhouxs@nuc.edu.cn

**Keywords:** intercritical quenching, banded microstructure, carbide precipitation, delamination fracture

## Abstract

The effects of intercritical quenching on the microstructure evolution and mechanical performance of Cr–Ni–Mo–V steel with a banded structure are studied. It is found that the intercritical quenching temperature has a significant effect on the morphology, distribution, and relative amount of ferrite/martensite, as well as the carbide precipitates upon tempering treatment. It is indicated that owing to the initial banded structure of Cr-Ni-Mo-V steel, the ferrite formation in intercritical heat treatment also exhibits a banded distribution. With the increase in quenching temperature, the proportion of ferrite in the Cr-Ni-Mo-V steel decreases from 30 ± 3.2 vol.% to 18 ± 2.8 vol.%. Tempering treatment has no significant effect on the distribution characteristics of ferrite, but it promotes the recovery of martensite laths and the precipitation of carbides. The mechanical properties of Cr-Ni-Mo-V steel are determined by both the changes in ferrite content induced by intercritical quenching and the evolution of carbide types during tempering. Delamination cracks are observed on the fracture surface, which is attributed to the lamellar microstructure, improving the plasticity of Cr-Ni-Mo-V steel through stress dispersion and a multi-stage energy absorption mechanism.

## 1. Introduction

Cr-Ni-Mo-V steels have been widely applied in the manufacturing of various engineering constructions, such as artillery barrels, high-pressure vessels, and steam turbine shafts, owing to their outstanding hardenability and an optimal balance of strength and toughness [1,2,3,4]. These components typically operate in harsh environments subjected to extreme conditions (e.g., high pressure and cyclic loading), where superior comprehensive mechanical properties are critical to ensuring service safety [5,6,7,8]. The addition of alloying elements, including Mn, Cr, Ni, Mo, and V, not only enhances the strength but also improves austenite stability, thereby significantly increasing the steel’s hardenability [9,10,11,12,13]. However, chemical segregation of alloying elements (e.g., Mn, V, and Cr) during casting can promote the formation of a banded microstructure, which in turn influences phase transformation behavior, carbide precipitation within the bands, and mechanical properties across these regions [14,15]. Furthermore, processes such as welding, additive manufacturing, and repair operations frequently introduce uncontrolled and detrimental microstructures in high-strength steels, including coarse martensite, residual stress concentrations, or localized segregation. Heat treatment, by contrast, acts as a critical corrective measure, effectively refining these problematic microstructures and optimizing the mechanical performance of the resulting martensitic steel components [16,17].

Quenching and tempering (Q&T) treatments are commonly applied to Cr-Ni-Mo-V steels, as they effectively enhance microstructural homogeneity [18,19,20,21,22,23,24]. However, this approach can only improve mechanical properties within a limited range and fails to overcome the strength–ductility trade-off. For low-alloy high-strength steels, intercritical heat treatment can serve as an alternative to conventional quenching and tempering processes. Deng et al. successfully prepared dual-phase (ferrite–martensite) steel by cold rolling and intercritical annealing, achieving a tensile strength of 866 MPa and a total elongation of 24.40% [25]. Wu et al. developed a lamellar heterostructured steel by introducing a soft δ-ferrite phase into a hard martensitic matrix, which exhibited simultaneous enhancements in both strength and ductility compared to conventional fully martensitic steel [26]. Sang et al. optimized the intercritical quenching and tempering process for medium-carbon steel to obtain a heterostructure, which showed superior mechanical properties over conventional pearlitic 4340 steel [27]. Gao et al. conducted warm rolling at 600 °C followed by short-time intercritical annealing on a ferrite–pearlite microstructure, thereby successfully producing lamellar ferrite–martensite dual-phase steel. This material achieved a significant strength improvement (up to 1.6 GPa) along with a high uniform elongation of 7% [28].

Previous studies have primarily focused on improving the mechanical performance of steels through the combination of rolling deformation and intercritical treatment, whereas limited attention has been devoted to investigating the effects of intercritical quenching on the microstructural evolution and mechanical properties of Cr-Ni-Mo-V steel with a banded structure. In the present work, we systematically examine the microstructural evolution and mechanical performance of Cr-Ni-Mo-V steel with a banded structure under varying intercritical quenching temperatures. A laminated heterostructure composed of ferrite (soft phase) and martensite (hard phase) is obtained, and the morphology, relative amount of ferrite and martensite, as well as carbide precipitation behavior, are discussed to explore their influence on the mechanical performance of the steel.

## 2. Materials and Methods

**Figure 1:**
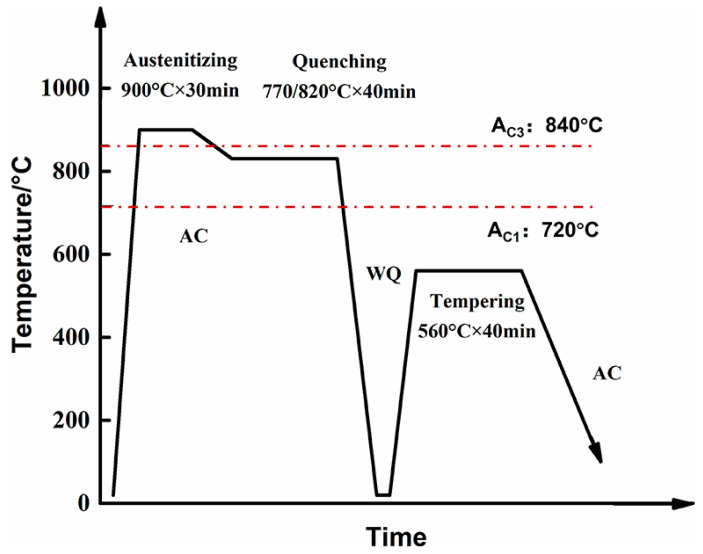
Schematic diagram of multi-step heat treatment process.

The material used in this study was a forged and annealed Cr-Ni-Mo-V steel, with its chemical composition listed in Table 1. The chemical composition listed in Table 1 was provided by the material supplier, and the chemical composition was determined by the inductively coupled plasma-atomic emission spectrometer (ICP-AES) using PerkinElmer Optima 8000 ICP-AES (PerkinElmer, Waltham, MA, USA). The austenite transformation temperatures of the experimental steel were determined using a differential scanning calorimeter of DSC 214 Polyma (Netzsch, Bremen, Germany), yielding Ac_1_ (the lower critical temperature at which austenite starts to form during heating) = 720 °C and Ac_3_ (the upper critical temperature at which austenite fully forms during heating) = 840 °C. The intercritical heat treatment for the experimental steel was performed as follows. First, the specimens were heated to 900 °C for 1 h to achieve full austenitization and were then slowly cooled to the two-phase region (770–820 °C) and held for 40 min, followed by water quenching in a water bath to room temperature. Subsequently, the quenched specimens were tempered at 560 °C for 40 min and then air-cooled to room temperature. It should be noted that for larger-scale engineering components, extended holding times during heat treatment are necessary to ensure uniform temperature distribution throughout the entire material volume, thereby avoiding microstructural inhomogeneity caused by temperature gradients. A schematic diagram of the heat treatment process for the investigated Cr-Ni-Mo-V steel is presented in Figure 1. Specimens intended for microstructural observation and mechanical properties testing were cut along a direction parallel to the forging direction.

Microstructural observations were performed using an optical microscope (OM, Zeiss Axio Imager. A2m, Carl Zeiss AG, Oberkochen, Germany), a scanning electron microscope (SEM, Hitachi-SU5000, Hitachi, Tokyo, Japan), and transmission electron microscopy (TEM, JEOL 2100F, JEOL, Tokyo, Japan). Specimens for microstructural characterization were prepared using standard metallographic procedures and etched with a 4% nital solution. TEM specimens were prepared by twin-jet electropolishing of (DJ2000, Beijing Dedong Technology Co., Ltd., Beijing, China) using a mixture of 90 vol.% ethanol and 10 vol.% perchloric acid. To quantitatively evaluate the proportion of ferrite/martensite in the microstructure, at least five SEM micrographs were processed by using the software ImageJ 1.53t. Repeated tensile tests were conducted on dog-bone-shaped specimens at room temperature using an Instron 3382 testing machine (Instron, Norwood, MA, USA), following the ASTM E8M standard [30]. Tensile specimens were machined into dog-bone shapes with a gauge length of 25 mm and a gauge diameter of 5 mm. The Vickers hardness was measured using an HR-320MS hardness tester (Mitutoyo, Nagoya, Japan) with a load of 500 gf and a dwell time of 15 s. For each sample, at least 10 indentations were distributed uniformly across the polished surface to account for potential microstructural inhomogeneity. The hardness value (HV) for each specimen was reported as the average of all valid measurements.

## 3. Results and Discussion

### 3.1. Quenched State

**Figure 2:**
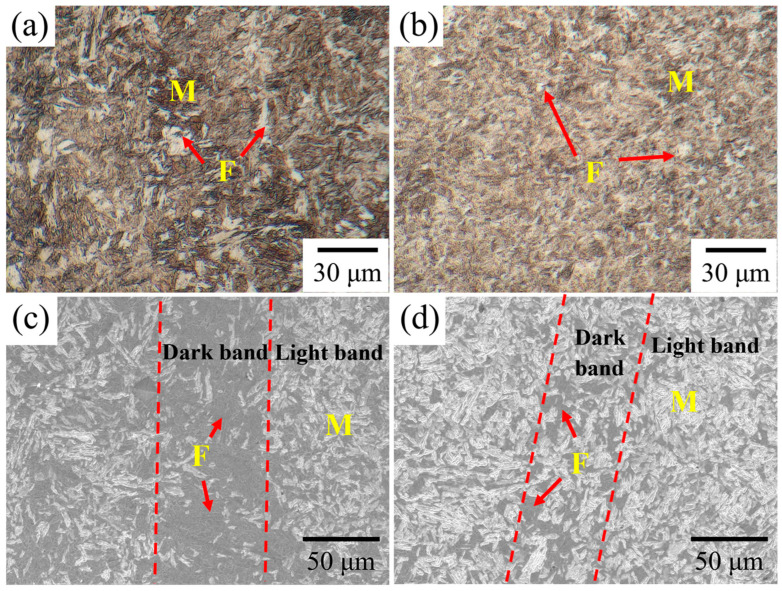
Microstructure of the Cr-Ni-Mo-V steel from the quenching temperature of (**a**,**c**) 770 °C and (**b**,**d**) 820 °C. M denotes martensite and F denotes ferrite. The dashed line indicates the interface between the light and dark bands.

The microstructure of Cr-Ni-Mo-V steel quenched at different intercritical temperatures is shown in Figure 2. As shown in Figure 2a, the quenched microstructure of Cr-Ni-Mo-V steel consists of a mixture of martensite and ferrite. Specifically, at a quenching temperature of 770 °C, a large amount of blocky ferrite is dispersedly distributed in the martensite matrix, as indicated by the red arrows in Figure 2a, with their volume fraction being significantly higher than that of 820 °C. As the quenching temperature increases to 820 °C, the large blocky ferrite virtually vanishes, and the acicular ferrite is observed distributed within the martensite matrix, as shown in Figure 2b. Figure 2c,d illustrate the SEM micrographs of Cr-Ni-Mo-V steel quenched at different temperatures. As shown in Figure 2c,d, the steel exhibits a mixed microstructure consisting of martensite and ferrite, with light and dark bands alternately distributed along the forging direction. For the steel quenched from 770 °C (Figure 2c), the volume fraction of ferrite in the dark band is higher than that in the light band. As shown in Figure 2d, after quenching from 820 °C, the bright band primarily comprises lath martensite with a small amount of ferrite distributed between the martensite laths. The volume fraction of ferrite in the dark bands is significantly lower than that quenching from 770 °C, but remains higher than that in the bright band. With the quenching temperature increasing from 770 °C to 820 °C, the volume fraction of ferrite in the microstructure decreases while the amount of martensite increases, and the morphology of martensite gradually transforms from discrete laths to bundled laths.

Statistical analysis conducted by ImageJ reveals that the volume fractions of ferrite at 770 °C and 820 °C are 30 ± 3.2 vol.% and 18 ± 2.8 vol.%, respectively, which is consistent with the experimental results. It can be seen that the increase in intercritical temperature leads to a decrease in the amount of ferrite and an increase in martensite, due to the larger driving force of ferrite transformation at a lower temperature. Thermodynamically, the lower the intercritical quenching temperature in the two-phase region, the more austenite transforms into ferrite, resulting in an increase in the ferrite content. The Gibbs free energy difference between austenite and ferrite is increased as the intercritical quenching temperature decreases. The ferrite formation is accompanied by carbide precipitation. With the carbide precipitation, the carbon supersaturated in martensite will be reduced. Moreover, the austenite grain size also has a significant effect on martensite formation. It is suggested that both the carbon content in martensite and prior austenite grain size are responsible for the martensite morphology change under different intercritical quenching temperatures.

**Figure 3:**
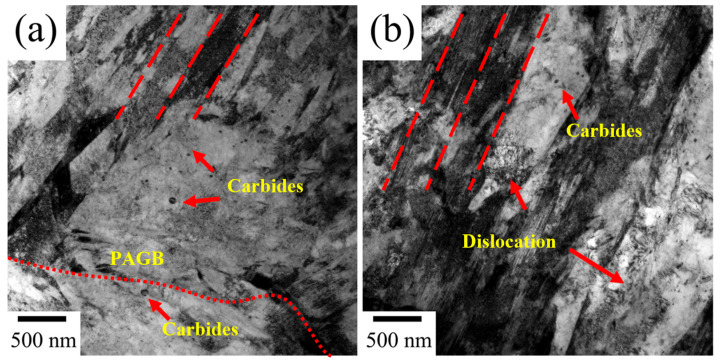
TEM micrographs of the Cr-Ni-Mo-V steel quenched from (**a**) 770 °C and (**b**) 820 °C, showing martensite laths. The dashed line indicates the martensite lath boundary.

Figure 3 shows the microstructural characteristics of Cr-Ni-Mo-V steels quenched at different temperatures. Martensitic laths with a high density of dislocations can be readily found. With the quenching temperature increased from 770 °C to 820 °C, the averaged lath width increases from 0.33 μm to 0.46 μm, which should be related to the larger size of austenite grains at 820 °C. It is interesting to note that nano-sized MC precipitates can be found within martensitic laths, irrespective of quenching temperature. These spherical MC carbides have high thermal stability, and it is difficult to dissolve them in the homogenization process [31]. Thus, MC carbides were retained in austenite and were then present in martensite due to martensitic transformation.

**Figure 4:**
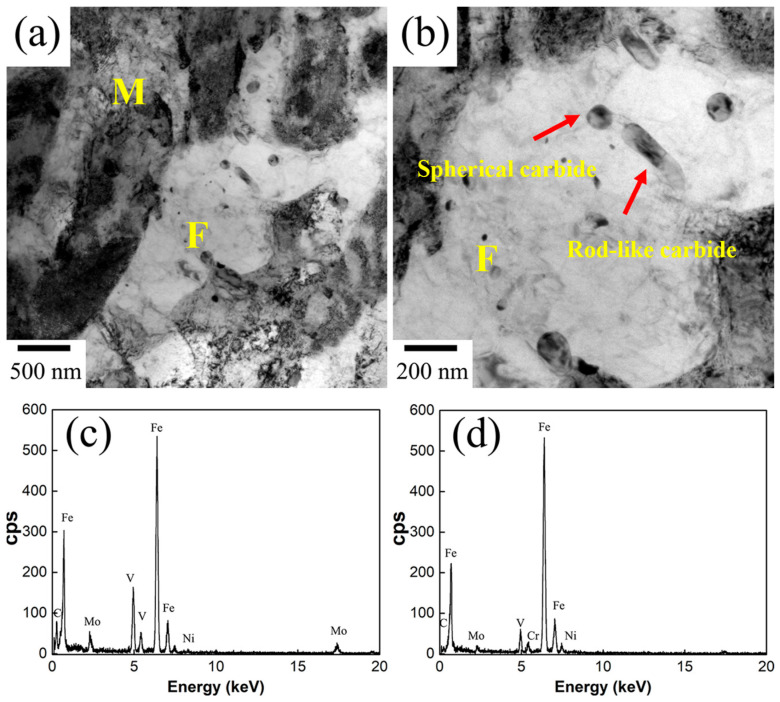
(**a**,**b**) TEM micrographs of the Cr-Ni-Mo-V steel quenched from the temperature of 770 °C; (**c**) EDS result of the spherical carbides, and (**d**) EDS result of the rod-like carbides.

**Figure 5:**
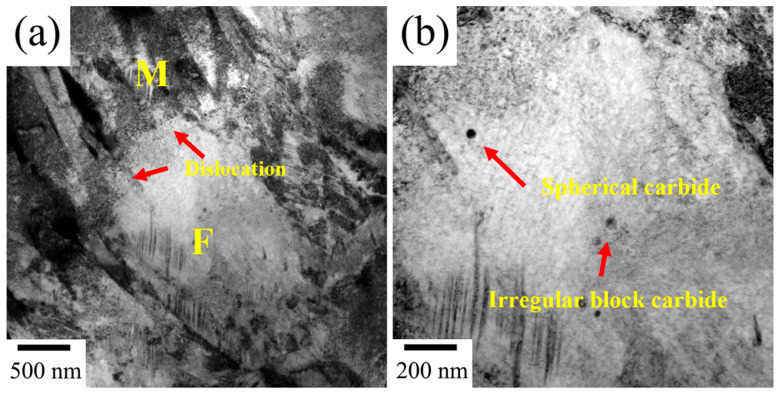
(**a**,**b**) TEM micrographs of the Cr-Ni-Mo-V steel quenched from the temperature of 820 °C.

TEM images of ferrite in Cr-Ni-Mo-V steel quenched from 770 °C are shown in Figure 4. It can be observed that a certain amount of spherical carbides and rod-like carbides are dispersedly distributed within the ferrite matrix or at the interface between martensite and ferrite. According to the EDS results shown in Figure 4c, the spherical carbides are mainly composed of Fe, C, V, Mo, and Ni, and these small-sized spherical carbides can be identified as MC carbides. The EDS result of the rod-like precipitate indicated in Figure 4b is shown in Figure 4d, and it should be the M_23_C_6_ carbides, exhibiting much larger sizes than the MC carbides. Different from the carbide precipitation in ferrite at 770 °C, as the intercritical temperature increases to 820 °C, only MC carbides are found to be precipitated within the ferrite, as shown in Figure 5. On the other hand, at the interface between the martensite and ferrite, a certain amount of dislocations can be found in the ferrite. It can be seen that the intercritical temperature has a significant effect on the carbide precipitation in ferrite transformation. As the intercritical temperature decreases, M_23_C_6_ carbides tend to precipitate in ferrite formation. At 820 °C, more austenite grains are retained, and then the volume expansion upon martensitic transformation is larger. The ferrite surrounded by the martensite will experience deformation, and then dislocations are generated within the ferrite close to the interface between ferrite and martensite.

### 3.2. Tempered State

**Figure 6:**
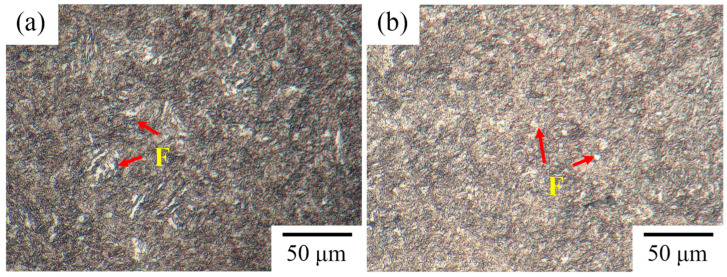
Optical micrographs of Cr-Ni-Mo-V steel after tempering process: (**a**) 770–560 °C; (**b**) 820–560 °C. F denotes ferrite.

**Figure 7:**
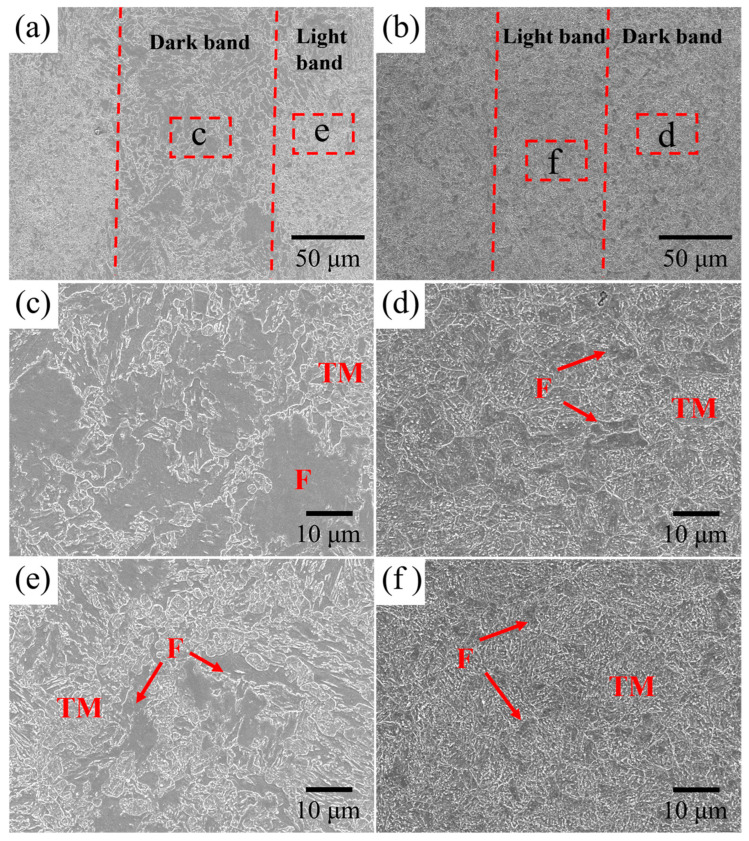
SEM micrographs of the Cr-Ni-Mo-V steel after tempering process: (**a**,**c**,**e**) 770–560 °C; (**b**,**d**,**f**) 820–560 °C. TM denotes tempered martensite and F denotes ferrite. The dashed line indicates the interface between the light and dark bands.

To improve the ductility, the Cr-Ni-Mo-V steel with different intercritical temperatures was subjected to tempering heat treatment. Figure 6 shows the optical micrographs of the tempered Cr-Ni-Mo-V steel. Tempering treatment has negligible effects on the ferrite distributions, and the ferrite can still be readily distinguished. As shown in Figure 7, the banded structure of Cr-Ni-Mo-V steel is still present after tempering. The ferrite distribution is related to the banded structure, as is the case in the quenched condition. From Figure 7, it can be seen that carbides are precipitated along lath boundaries, prior austenite grain boundaries, and the interfaces between ferrite and martensite.

**Figure 8:**
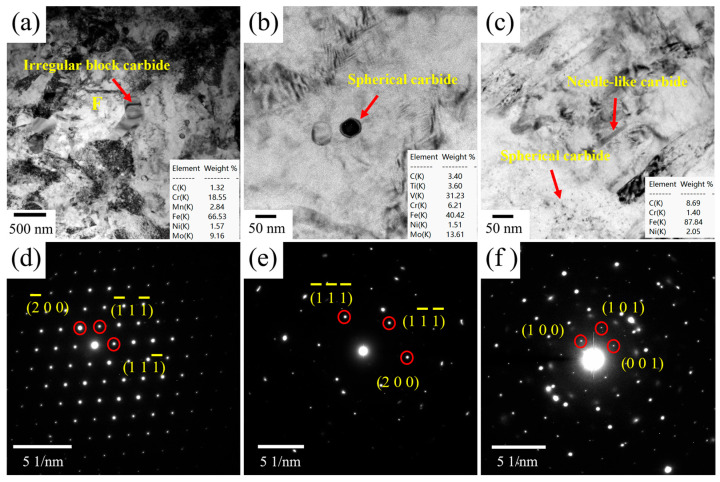
(**a**–**c**) TEM micrograph of tempered Cr-Ni-Mo-V steel quenched from 770 °C; (**d**–**f**) corresponding SAD pattern of the irregular block, spherical, and needle-like carbides.

**Figure 9:**
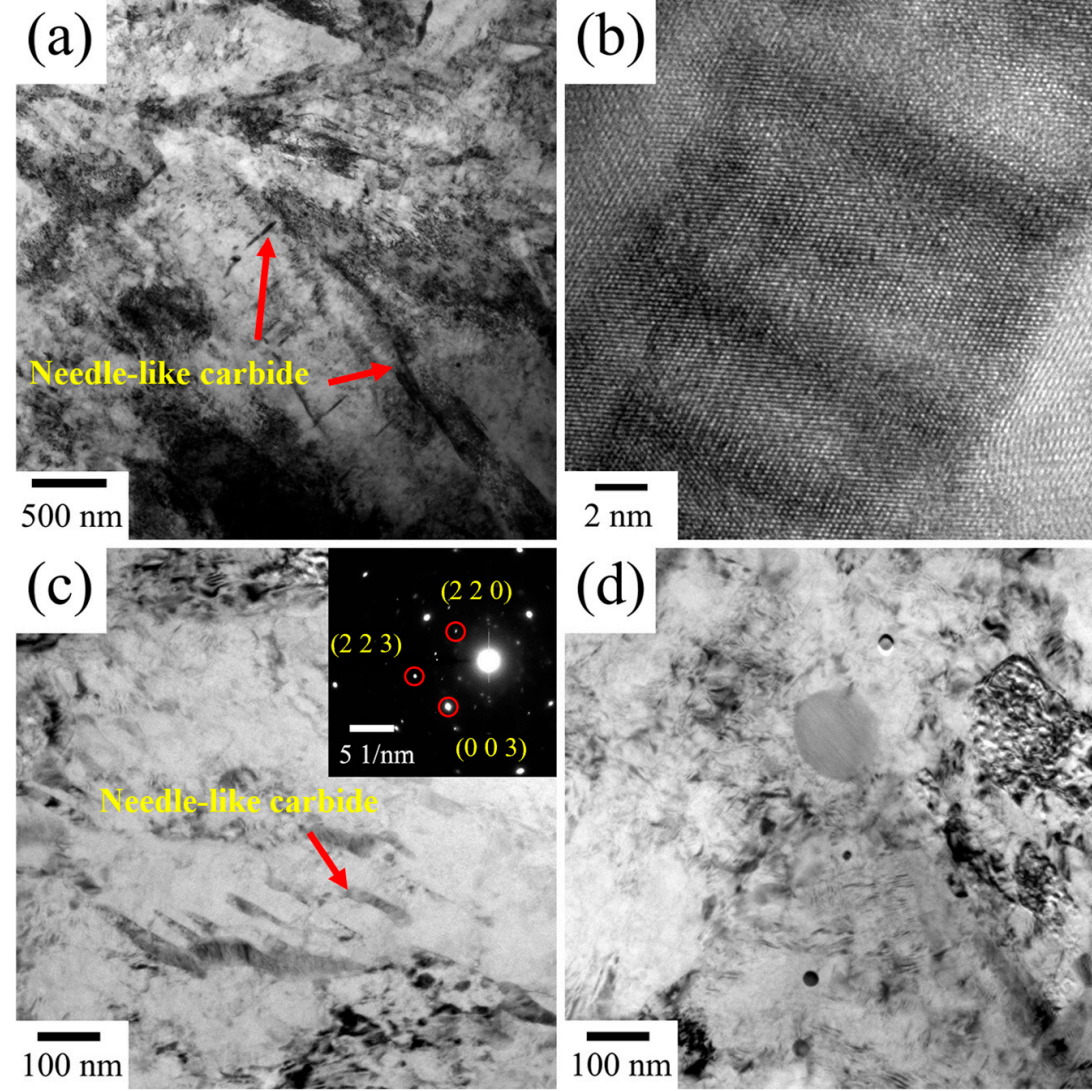
TEM micrograph of tempered Cr-Ni-Mo-V steel quenched from 820 °C, showing (**a**) needle-like M_3_C, (**c**) strip-like M_3_C, and (**d**) nano-sized MC. (**b**) HR-TEM image of the needle-like carbide.

TEM images of tempered Cr-Ni-Mo-V steel with an intercritical temperature of 770 °C are shown in Figure 8. During tempering, the coarsening of martensite laths occurs due to the gradual reduction in dislocations as lath martensite undergoes recovery. This process is accompanied by the recombination of sub-grain boundaries, ultimately leading to the disappearance of lath boundaries. Meanwhile, compared with the quenched microstructure, the dislocation pile-ups within ferrite grains and at grain boundaries are significantly relieved. As shown in Figure 8a, some large blocky carbides with a size of ~80 nm are distributed at grain boundaries. Based on the SAD pattern and EDS result in Figure 8a,d, these irregular block carbides are identified as M_23_C_6_ carbides. Spherical carbides with an average diameter of approximately 43 nm can be observed in Figure 8b, and these large-sized spherical carbides are identified as MC carbides based on the SAD pattern and EDS result. In Figure 8c, a high density of nano-sized spherical carbides (~5 nm in diameter) is distributed within tempered martensite. It is suggested that the large-sized MC shown in Figure 8b should be undissolved MC in the intercritical heat treatment, while the small-sized MC carbides are precipitated from martensite upon tempering, which is in agreement with previous work [2,32]. Except for small-sized MC, needle-like M_3_C carbides are also formed in tempered martensite, as indicated in Figure 8c. Figure 9 shows the TEM images of tempered Cr-Ni-Mo-V steel with an intercritical temperature of 820 °C. In this case, needle-like and strip-like M_3_C carbides can be readily found within martensite, as shown in Figure 9a,c. Figure 9b shows the HRTEM image of the M_3_C carbide. Compared to an intercritical temperature of 770 °C, more M_3_C carbides are precipitated in martensite when the intercritical temperature is 820 °C, which is consistent with the carbide precipitation shown in Figure 7. Nano-sized MC carbides are also precipitated within laths, as shown in Figure 9d. It seems that the precipitation density of MC carbides under an intercritical temperature of 820 °C is smaller than that under 770 °C. The reduced distribution density of MC carbides at the quenching temperature of 820 °C results from the combined effects of thermodynamic dissolution and kinetic limitations. Their high solubility product enhances dissolution in austenite at this temperature, increasing V, Mo, and C solubility in the matrix and reducing precipitation drive. Meanwhile, faster atomic diffusion is offset by lower supersaturation of these elements, restricting MC nucleation. These factors collectively result in fewer MC carbides at 820 °C compared to 770 °C.

### 3.3. Mechanical Properties and Deformation Behavior

**Figure 10:**
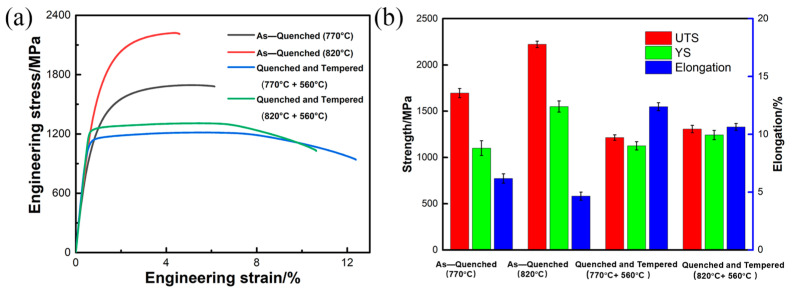
Mechanical properties of the Cr-Ni-Mo-V steel subjected to various heat treatment processes: (**a**) engineering stress–strain curves and (**b**) corresponding tensile strength, yield strength, and elongation.

Figure 10a presents the engineering stress–strain curves at room temperature for samples subjected to various heat treatment processes, while Figure 10b summarizes the tensile strength, yield strength, and elongation of the steels. In the quenched state, the increase in intercritical quenching temperature from 770 °C to 820 °C results in a significant increase in tensile strength from 1695 MPa to 2221 MPa, accompanied by a decrease in elongation from 6.1% to 4.6%. The mechanical properties of Cr-Ni-Mo-V steel are significantly affected by the dual-phase microstructure of ferrite and martensite with a varying amount, which is closely related to the intercritical quenching temperature. For the specimen quenched from 770 °C, which contains 30 ± 3.2 vol.% ferrite, the higher fraction of this soft phase contributes to better ductility (an elongation of 6.1%), while the relatively lower martensite content results in a tensile strength of 1695 MPa. Extensive studies confirm that increased martensite content induces higher residual stresses and elevated dislocation density in neighboring ferrite, substantially strengthening dual-phase steels [27,33]. In contrast, the specimen quenched from 820 °C, where the ferrite content decreases to 18 ± 2.8 vol.%, exhibits significantly enhanced strength (2221 MPa) due to the increased martensite proportion. However, the reduction in the ferrite amount leads to a decrease in elongation to 4.6%. It is suggested that the tensile strength is closely related to the martensite characteristics under different intercritical quenching temperatures. More martensite will contribute to the increase in strength.

During the tempering process, the coupled effects of carbide precipitation, dislocation density reduction, and martensite lath coarsening contribute to a moderate decrease in strength accompanied by improved ductility. Specifically, the tempered steel with an intercritical temperature of 770 °C exhibits a tensile strength of 1214 MPa with an elongation of 12.4%, whereas the tempered steel with an intercritical temperature of 820 °C shows a higher strength of 1307 MPa but a lower elongation of 10.7%. After tempering at 560 °C, the evolution of carbide types and their distribution emerges as a critical factor influencing mechanical properties. Under an intercritical temperature of 770 °C, the synergistic effect of multiple carbides (MC, M_23_C_6_, and M_3_C), combined with martensite decomposition and dislocation density reduction, results in a moderate tensile strength decrease to 1214 MPa while achieving a remarkable elongation enhancement to 12.4%, thus demonstrating a superior strength–ductility balance. In contrast, for the 820 °C intercritical treatment, large-sized M_23_C_6_ carbides are absent, and a higher density of M_3_C carbides precipitates, contributing to the higher strength observed. After the tempering heat treatment, the tempered steel quenched from 820 °C has a higher hardness (385 ± 5 HV) than the tempered steel quenched from 770 °C (345 ± 8 HV), which is consistent with the tensile results. It is noteworthy that the strength difference between the two tempered conditions is much smaller than that in the quenched state, which is attributed to the reduced hardness of martensite upon tempering [34,35,36].

In quenched conditions, the carbon atoms supersaturated in martensite will lead to a strong solid-solution strengthening effect. Upon tempering, the carbides are formed, and the precipitation strengthening effect is enhanced, but the solid-solution strengthening effect is weakened. With respect to a quenching temperature of 770 °C, since more ferrite is formed and more carbides are precipitated within the ferrite, the solid-solution strengthening effect caused by carbon in quenched martensite is not as strong as that under 820 °C, and tempering heat treatment has a smaller effect on the hardness difference between quenched martensite and tempered martensite. It can also be found that the strength difference between the tempered steel of 820 °C and the tempered one of 770 °C is smaller than that between the quenched steels. This means that the stronger solid-solution strengthening effect of supersaturated carbon on martensite in quenched steel at 820 °C is substantially reduced upon tempering. Although more carbides are precipitated in the steel with a quenching temperature of 820 °C, the strength increase caused by the carbide precipitation cannot overwhelmingly offset the strength decrease caused by the reduction in solid solubility of carbon in martensite. With respect to the steel quenched at 770 °C, the carbon atoms supersaturated with the martensite are less than those in the quenched steel at 820 °C, due to the carbide formation upon ferrite transformation. Then, tempering has a smaller effect on the strength contribution from solid-solution strengthening. The tempered steel quenched from 820 °C exhibits slightly higher strength than the tempered steel quenched from 770 °C.

**Figure 11:**
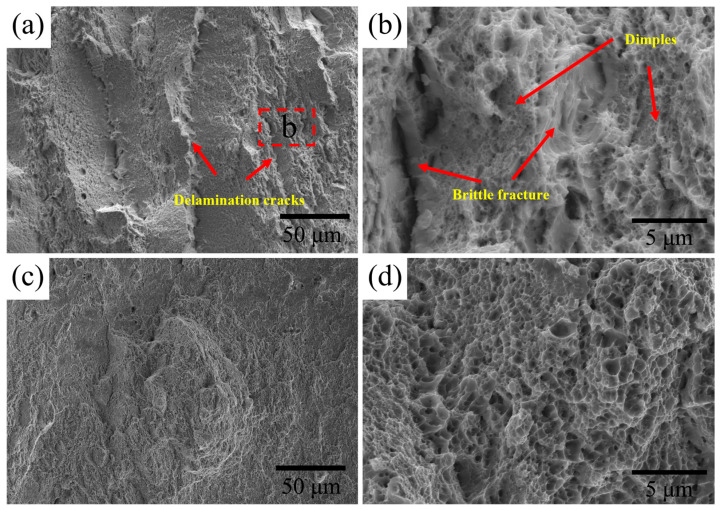
SEM images of the tensile fracture surface of the tempered steel at the intercritical temperature of (**a**,**b**) 770 °C and (**c**,**d**) 820 °C.

Figure 11 presents the SEM images of the tensile fracture morphology of tempered Cr-Ni-Mo-V steel with different intercritical temperatures. At the intercritical temperature of 770 °C, as shown in Figure 11a, delamination cracks with varying lengths are observed along the thickness direction. Further analysis of the delamination cracks, as illustrated in Figure 11b, reveals that the initiation sites of the delamination cracks are relatively smooth, characterized by distinct river patterns and tear ridges, thus exhibiting brittle fracture characteristics. Notably, the matrix region far from the layered interfaces contains numerous dimples with an average diameter of approximately 2–3 μm, confirming that this region undergoes a ductile fracture via a microvoid coalescence mechanism. This classical failure mechanism proceeds in three sequential stages: nucleation of microvoids at stress concentrators, void growth under plastic deformation, and finally, coalescence of macroscopic cracks [37,38]. In contrast, under the intercritical temperature of 820 °C, no obvious delamination cracks or tear ridges are observed, and the dimple sizes are more homogeneous, as shown in Figure 11c,d. Due to the initial banded structure of Cr-Ni-Mo-V steel, the ferrite formation in intercritical heat treatment also exhibits a banded distribution, especially at the intercritical temperature of 770 °C. During tensile loading, the soft regions (ferrite) preferentially undergo plastic flow, leading to stress concentration at the phase boundaries; when the strain reaches a critical value, the inhomogeneous deformation between the hard regions (martensite) and soft regions triggers interface separation, resulting in the formation of initial cracks at the interfaces of the layered ferrite and martensite, which then propagate along these interfaces. This initial crack formation and propagation process establishes the basis for subsequent energy absorption, as delamination cracks commence their role in regulating stress distribution. From the obvious delamination cracks shown in Figure 11a,b, it can also be inferred that banded ferrite–martensite structures tend to induce stress concentration at phase boundaries under fatigue loading, thereby promoting the initiation of fatigue cracks. Regarding carbides, fine and uniformly distributed carbides like MC are beneficial to retard crack propagation through dislocation pinning, whereas coarse or clustered carbides like M_3_C should act as preferential nucleation sites for fatigue cracks.

The formation and propagation of delamination cracks further mitigate overall stress concentration by dispersing localized stress to multiple interfacial regions, thereby preventing a sudden fracture induced by rapid stress accumulation at a single location, as documented in other lamellar-structured steels [15,39]. This stress dispersion effect endows Cr-Ni-Mo-V steel with more time for plastic deformation and enables higher strain accumulation prior to fracture. In addition, through a multi-stage energy absorption mechanism, delamination cracks first reduce the local stress intensity around the main crack tip; subsequently, their interaction with the main crack induces crack tip blunting and generates a layered fracture morphology via merging; finally, delamination cracks propagate along interfaces ahead of the main crack, with each propagation event dissipating energy incrementally. This progressive fracture behavior, coupled with the repeated initiation, propagation, and blunting of the main crack, delays final failure and significantly enhances overall ductility, thereby contributing to the improved plasticity of the Cr-Ni-Mo-V steel [40,41].

## 4. Conclusions

This work systematically investigated the effects of different intercritical quenching temperatures on the microstructural evolution, carbide precipitation behavior, and mechanical properties of Cr-Ni-Mo-V steel with a banded structure. The main conclusions are as follows:

(1) Intercritical quenching temperature has a significant effect on the morphology, distribution, and relative amount of ferrite/martensite of Cr-Ni-Mo-V steel. At the intercritical temperature of 770 °C, 30 ± 3.2 vol.% ferrite with a blocky morphology is obtained; as the quenching temperature increases to 820 °C, the amount of ferrite in the Cr-Ni-Mo-V steel decreases to 18 ± 2.8 vol.%.
(2) Tempering treatment has no significant effect on the distribution characteristics of ferrite, but it promotes the recovery of martensite laths and the precipitation of carbides. Specifically, under a quenching temperature of 770 °C, carbides precipitated upon tempering, including MC, M_23_C_6_, and M_3_C. Under a quenching temperature of 820 °C, the precipitation of carbides is mainly M_3_C, and the distribution density of MC is reduced.
(3) The mechanical properties of Cr-Ni-Mo-V steel are determined by both the variation in ferrite content induced by intercritical quenching and the evolution of carbide types during tempering. Delamination cracks observed on the fracture surface improve the ductility of Cr-Ni-Mo-V steel through stress dispersion and a multi-stage energy absorption mechanism.

## Figures and Tables

**Table 1:** Contents of various alloying elements in the studied steel (wt.%) [29].

C	Si	Mn	Cr	Ni	Mo	Cu	V	P	S	Fe
0.36	0.27	0.32	1.3	3.19	0.4	0.12	0.2	0.005	0.001	Bal.

## Data Availability

The original contributions presented in this study are included in the article material. Further inquiries can be directed to the corresponding author.
